# Angiotensin II and EDH Pathways Underlie the Vascular Sympatho-Modulation by 5-HT in Female Rats

**DOI:** 10.3390/ijms26199614

**Published:** 2025-10-01

**Authors:** Anaïs Clara Terol-Úbeda, Juan Francisco Fernández-González, Asunción Morán, Mónica García-Domingo, José Ángel García-Pedraza

**Affiliations:** 1 Laboratorio de Farmacología, Departamento de Fisiología y Farmacología, Facultad de Farmacia, Universidad de Salamanca, 37007 Salamanca, Spain; aniter99@usal.es (A.C.T.-Ú.); joseagp@usal.es (J.Á.G.-P.); 2 Instituto de Investigación Biomédica de Salamanca (IBSAL), Paseo San Vicente 58-182, 37007 Salamanca, Spain

**Keywords:** 5-HT_1D_ and 5-HT_2A_ receptors, angiotensin II, female rats, K^+^ channels, sympatho-modulation, vasopressor responses

## Abstract

The vascular 5-HT sympatho-modulation may involve inhibitory or potentiating pathways: nitric oxide (NO), endothelium-dependent hyperpolarization (EDH)-K^+^ channels, prostanoids, angiotensin II (Ang-II), or endothelin. Compared to males, female rats show differences in the serotonergic sympatho-regulation; therefore, we aimed to study the involvement of indirect pathways via 5-HT_1D_-mediated inhibition and 5-HT_2A/3_-mediated potentiation of vascular noradrenergic neurotransmission in females. An i.v. bolus of different inhibitors/blockers of modulators/mediators (NO, K^+^ channels, prostanoids, Ang-II, or endothelin) was administered prior to the infusion of the agonists, L-694,247 (5-HT_1D_), TCB-2 (5-HT_2A_), or 1-PBG (5-HT_3_), in female pithed rats. In these conditions, the vascular sympathetic outflow was electrically stimulated to assess the vasopressor responses. The L-694,247 vascular sympatho-inhibition was abolished by a non-selective K^+^ channel blocker, tetraethylammonium. The 1-PBG sympatho-excitatory vascular effect was not modified by any of the inhibitors tested, whereas TCB-2 sympatho-potentiation was blocked solely by losartan (Ang-II type 1 receptor antagonist). Moreover, Ang-II levels were increased after TCB-2 infusion in females. The EDH pathway mediates the 5-HT_1D_-induced sympatho-inhibition, while the 5-HT_2A_-evoked sympatho-excitatory effect is associated with Ang-II. In contrast, the 5-HT_3_ sympatho-potentiation does not involve any indirect pathway. These findings advance current understanding of the complex interactions between 5-HT and vascular homeostasis in female rats.

## 1. Introduction

The serotonergic system is well known as a cardiovascular modulator, acting not only directly on blood vessels [1,2,3,4] but also indirectly, by regulating other systems, such as the sympathetic system, the cholinergic system, or the non-adrenergic-non-cholinergic system in male rats [5,6,7,8,9,10].

Recent evidence suggests that sex differences in the serotonergic axis may contribute to different 5-hydroxytryptamine (5-HT, serotonin) pathophysiological regulation at the central and peripheral level [11,12,13], possibly due to different rates of serotonin synthesis [14] or 5-HT receptor expression [15,16]. Although 5-HT within the central nervous system (CNS) helps prevent arterial blood pressure increases in both sexes, this effect is more pronounced in males than in females, possibly due to ovarian hormones [17]. At the peripheral level, our group has recently shown that 5-HT contribution to cardiovascular homeostasis is also influenced by sex. In female rats, we have demonstrated that 5-HT_1D_ activation exerts a vascular sympatho-inhibitory action, as it does in males [6,7,18]; in contrast, while in male rats the sympatho-enhancing effect at the vascular level is mediated exclusively by 5-HT_3_ receptors [7,19,20], in females, the 5-HT_2A_ receptor also contributes to the increase in sympathetic-induced vasoconstrictions [18].

Cardiovascular disease (CVD) is the leading cause of mortality and premature death in women worldwide. Traditional cardiovascular risk factors manifest differently in women compared to men, and these differences have implications for management and clinical outcomes [21]. The mechanisms responsible for these sex differences are likely to be multifactorial, including clinical (e.g., blood vessels size), social (e.g., occupational hazards or habits), and biological aspects (which can be approached in animal models), such as estrogen or endothelial function/dysfunction.

The female sex hormone regulates vascular physiological features and function by modulating ion fluxes on smooth muscle cells and regulating endothelial-dependent vasodilator production and activity [22,23]. In fact, a previous report showed that stimulation of endothelial cells of female arteries results in significant endothelium-dependent hyperpolarization (EDH), which regulates blood pressure and may contribute to the lower incidence of CVD in premenopausal women [24]. In animal models, specifically in stroke-prone spontaneously hypertensive rats, female vessels are able to relax significantly more than male’s, probably due to higher expression or preserved function of ATP-sensitive potassium (K_ATP_) channels in female vessels [25].

The contribution of vasoconstrictor agents, such as endothelin or angiotensin II (Ang-II), to blood pressure regulation also seems to be sex-dependent. In this line, there are controversial results, as several authors reported that female rats had a greater blood pressure-lowering response to angiotensin-converting enzyme inhibition and a greater response to chronic Ang-II infusion than male rats [26,27], while others indicated that chronic Ang-II increases blood pressure to a higher extent in male than in female mice [28].

In male rats, our group has already shown in different experimental models that direct 5-HT effect on renal vasculature is linked to the Ang-II or the cyclooxygenase (COX) pathway [2,3] while the chronic 5-HT_2_ receptor blockade induces a vasodilator effect mediated by 5-HT_1D_, 5-HT_1B_, and 5-HT_7_ receptors, involving COX-derived prostacyclin, nitric oxide (NO) synthesis/release, and K_ATP_ channels, respectively [4]. Moreover, in males, serotonergic modulation of sympathetic outflow at the vascular level is also regulated by different vasoactive agents depending on the experimental model and the 5-HT receptor subtype involved such as NO, COX, or EDH [29,30,31]. However, to date, no evidence exists on the possible contribution of vasoactive agents (either vasorelaxants or vasoconstrictors) to the serotonergic modulation of vascular sympathetic outflow in females. Against this background, our hypothesis was that in female rats, vasoactive agents involved in serotonergic sympatho-modulation differ from males. Thus, the objective of this work was to determine the possible mechanisms involved in the sympatho-modulatory effect of the serotonergic system at the vascular level in female rats, evaluating different indirect vasoactive pathways (NO, COX, EDH pathways via K^+^ channels, endothelin, and Ang-II) that may be implicated in the vascular 5-HT_1D_-induced sympatho-inhibition and 5-HT_3_- and 5-HT_2A_-provoked sympatho-potentiation in females.

## 2. Results

### 2.1. Systemic Hemodynamic Parameters

**Figure 1:**
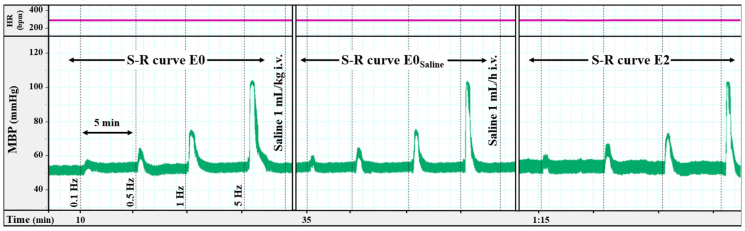
**Original experimental tracing showing the vasopressor responses induced by electrical sympathetic stimulation.** Electrically induced vasopressor responses and the effect of saline (1 mL/kg i.v. bolus followed by 1 mL/h i.v. infusion) in female pithed rats. Heart rate (HR; bpm) and mean blood pressure (MBP; mm Hg) are shown in the figure. S-R curves before saline administration (E0), after i.v. bolus of saline (E0_saline_) and during i.v. saline perfusion (E2) are represented. Notably, heart rate did not change significantly throughout the experiment. The vasopressor responses returned to baseline levels immediately after electrical stimulation. bpm: beats per minute; S-R: (electrical) stimulus-response.

The resting mean blood pressure (MBP) and heart rate (HR) in female pithed rats were 58 ± 1 mm Hg and 307 ± 4 beats/min (bpm), respectively. These values were not significantly altered following i.v. bolus administration of the vehicles (saline, Figure 1; or mixture of 33% polyethylene glycol, 33% ethanol, and 34% NaOH 0.2 M (PEN)), indomethacin or sulfisoxazole (Table 1), as previously reported in males [4,5,31]. Losartan induced a significant transient decrease in MBP (−12.4 ± 1.2 mm Hg) which returned to baseline within 10 min. In contrast, i.v. bolus administration of N(ω)-nitro-L-arginine methyl ester (L-NAME) resulted in a sustained increase in MBP, while tetraethylammonium (TEA) reduced HR (Table 1).

Intravenous infusion of saline (Figure 1) or L-694,247 (Table 1) did not affect baseline hemodynamic parameters. However, TCB-2 increased both MBP and HR, whereas 1-phenylbiguanide (1-PBG) selectively increased HR without altering MBP (Table 1). None of the antagonists modified the baseline hemodynamic values of the serotonergic agonists.

### 2.2. Effect of Saline or 5-HT Receptor Agonists: L-694,247, TCB-2, and 1-PBG on the Vasopressor Responses Induced by Electrical Stimulation in Female Rats

**Figure 2:**
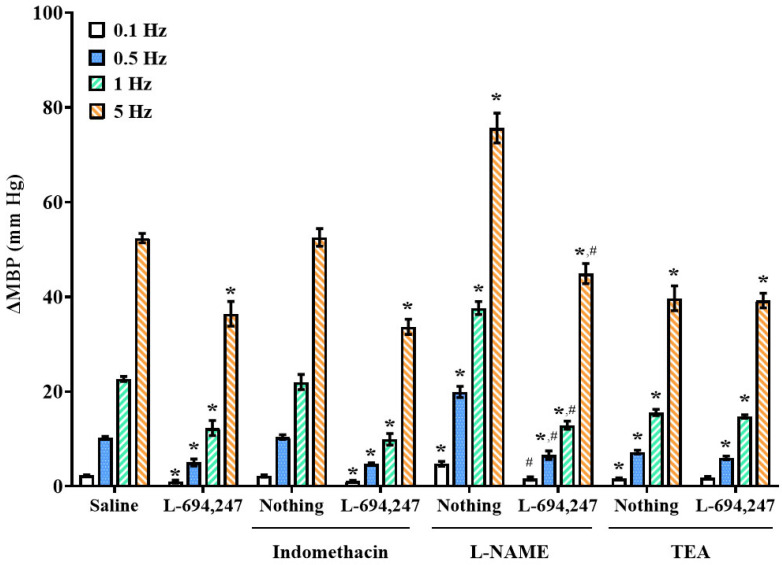
**Effect of the i.v. administration of different inhibitors/blockers of vasoactive mediators on the vascular sympatholytic effect induced by 5-HT_1D_ activation.** Increases in mean blood pressure (∆MBP; mm Hg) induced by electrical sympathetic stimulation during i.v. perfusion of saline (control; 1 mL/h) and i.v. perfusion of L-694,247 (10 µg/kg/min) in the absence or presence of i.v. bolus of indomethacin (2 mg/kg), TEA (16.5 mg/kg), or L-NAME (10 mg/kg) in female rats. Data were analyzed by two-way ANOVA followed by Dunnett’s post hoc test. All values are expressed as mean ± SEM. * *p* < 0.05 vs. saline. ^#^ *p* < 0.05 vs. L-694,247 alone. L-NAME: N(ω)-nitro-L-arginine methyl ester; TEA: tetraethylammonium.

**Figure 3:**
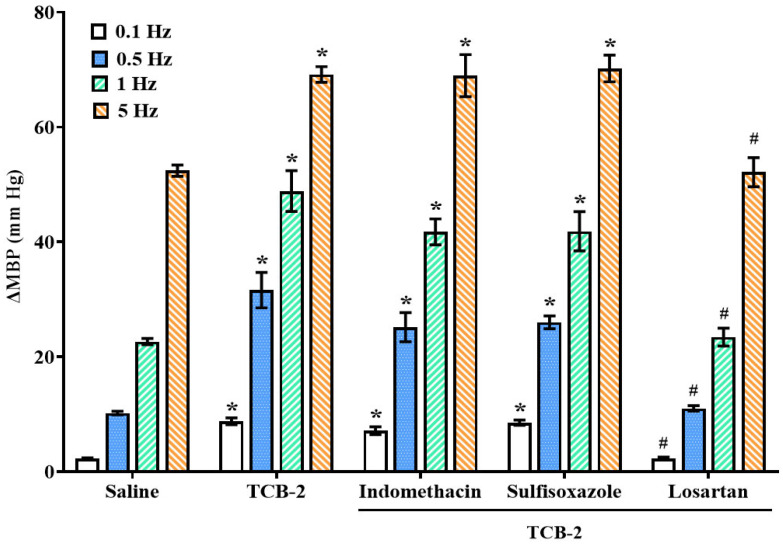
**Effect of the i.v. administration of different inhibitors/blockers of vasoactive agents on the vascular sympatho-enhancement triggered by 5-HT_2A_ activation.** Effect of the i.v. bolus of indomethacin (2 mg/kg), sulfisoxazole (0.5 mg/kg), or losartan (1 mg/kg) on the potentiating effect of TCB-2 (1 µg/kg/min) on the electrically induced vasopressor responses in female rats. Data were analyzed by two-way ANOVA followed by Dunnett’s post hoc test. All values are expressed as mean ± SEM. * *p* < 0.05 vs. saline; ^#^ *p* < 0.05 vs. TCB-2 alone. ∆MBP: increases in mean blood pressure.

**Figure 4:**
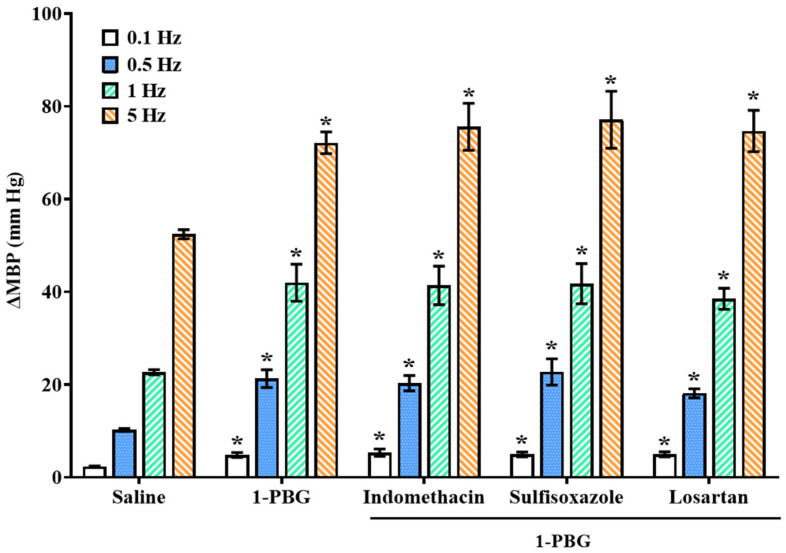
**Effect of the i.v. administration of different inhibitors/blockers of vasoactive substances on the vascular sympatho-potentiation evoked by 5-HT_3_ activation.** Effect of the i.v. bolus of indomethacin (2 mg/kg), sulfisoxazole (0.5 mg/kg), or losartan (1 mg/kg) on electrically induced vasoconstrictions in the presence of 1-PBG infusion (10 µg/kg/min) in female rats. Data were analyzed by two-way ANOVA followed by Dunnett’s post hoc test. All values are expressed as mean ± SEM. * *p* < 0.05 vs. saline. No statistical significance vs. 1-PBG alone. 1-PBG: 1-phenylbiguanide. ∆MBP: increases in mean blood pressure.

Electrical stimulation of vascular sympathetic nerves induced frequency-dependent increases in MBP (2.2 ± 0.3, 10.5 ± 0.5, 24.8 ± 2.3, and 54.1 ± 3.8 mm Hg for 0.1, 0.5, 1, and 5 Hz, respectively; control stimulation–response curve (S-R curve E0)). These vasopressor responses were not altered after i.v. infusion of saline (2.2 ± 0.2, 10.2 ± 0.3, 22.7 ± 0.5, and 52.4 ± 1.0, respectively; Figure 2, Figure 3 and Figure 4).

Continuous infusion of the selective 5-HT_1D_ agonist, L-694,247 (10 µg/kg/min), inhibited the sympathetic-induced vasopressor responses (Figure 2) [18]. In contrast, i.v. perfusion of the 5-HT_2A_ and 5-HT_3_ receptor agonists, TCB-2 (1 µg/kg/min) and 1-PBG (10 µg/kg/min), respectively, potentiated the electrically induced vasoconstrictions at all frequencies tested (Figure 3 and Figure 4).

### 2.3. Influence of i.v. Bolus of Vehicles or Antagonists (Indomethacin, L-NAME, TEA, Sulfisoxazole, or Losartan) per se on the Vasopressor Responses Induced by Electrical Stimulation in Female Rats

Intravenous bolus administration of vehicles (saline and PEN, 1 mL/kg each; Figure 1), indomethacin (a non-selective COX inhibitor; 2 mg/kg), sulfisoxazole (an endothelin ET_A_ receptor antagonist; 0.5 mg/kg), or losartan (an Ang-II type 1 (AT_1_) receptor antagonist; 1 mg/kg) did not significantly modify the S-R curve E0 (Table 2). However, i.v. administration of L-NAME (a non-selective NO synthase (NOS) inhibitor; 10 mg/kg) enhanced the vasopressor responses induced by electrical stimulation, whereas i.v. administration of TEA (a non-selective K^+^ channel blocker; 16.5 mg/kg) reduced the sympathetic-induced vasopressor responses (Table 2, Figure 2).

### 2.4. Impact of i.v. Administration of Indomethacin, L-NAME, or TEA on the L-694,247-Induced Serotonergic Sympatho-Inhibitory Effect in Female Rats

The inhibitory effect of L-694,247 (5-HT_1D_ receptor agonist) on the electrically induced vasoconstrictions was exclusively abolished by the i.v. administration of TEA (Figure 2). In contrast, the i.v. administration of indomethacin or L-NAME failed to reverse the inhibitory action of L-694,247 on the vasopressor responses induced by sympathetic stimulation in female rats (Figure 2).

### 2.5. Influence of i.v. Administration of Indomethacin, Sulfisoxazole, or Losartan on the TCB-2 or 1-PBG Sympathetic Potentiation in Female Rats

The i.v. pretreatment with indomethacin or sulfisoxazole did not alter the sympatho-potentiation induced by the 5-HT_2A_ receptor agonist, TCB-2; but, i.v. administration of losartan completely blocked the sympatho-potentiation induced by TCB-2 in female rats (Figure 3). The enhancing effect of 1-PBG (5-HT_3_ receptor agonist) on the vasopressor responses evoked by sympathetic stimulation was not modified by pretreatment with either indomethacin, sulfisoxazole, or losartan (Figure 4).

### 2.6. Plasma Concentration of Ang-II

Basal plasma concentration of Ang-II was similar between male and female pithed rats (29.5 ± 2.9 and 25.1 ± 2.5 pg/mL, respectively). After 25 min of TCB-2 (5-HT_2A_ receptor agonist) perfusion in female pithed rats, Ang-II levels significantly increased (38.6 ± 4.3 pg/mL; * *p* < 0.05 vs. basal Ang-II levels in female pithed rats).

## 3. Discussion

**Figure 5:**
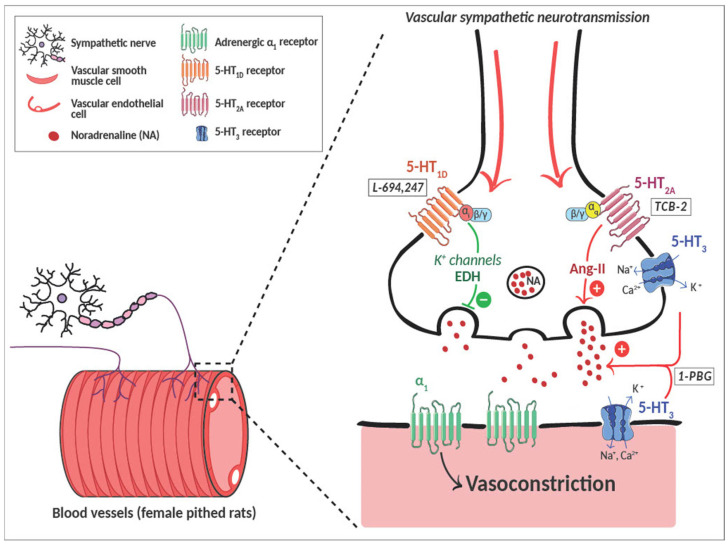
**Scheme summarizing the main outcomes in 5-HT modulation of the vascular sympathetic neurotransmission in female rats.** At the vascular level, L-694,247 (5-HT_1D_ agonist) sympatho-inhibition is mediated by the K^+^ channels opening, TCB-2 (5-HT_2A_ agonist) sympatho-excitatory action is produced via AT_1_ activation and 1-PBG (5-HT_3_ agonist) sympatho-potentiation is a direct effect in females. 1-PBG: 1-phenylbiguanide; Ang-II: angiotensin II; EDH: endothelium-dependent hyperpolarization.

Our study put in relevance, for the first time, that 5-HT_1D_ sympatho-inhibition at the vascular level is mediated by the K^+^ channels opening and the 5-HT_2A_ sympatho-excitatory effect is produced via AT_1_ activation in female rats. On the contrary, the 5-HT_3_ sympatho-modulatory effect seems to be direct in females (Figure 5).

### 3.1. Basal Hemodynamic Parameters in Female Rats

In our experiments, we used 14–16-week-old female rats, as representative of animals in fertile age [18]. Moreover, and continuing with our previous work, we utilized the model of the pithed rat [8,9,18,32], where MBP was around 58 mm Hg and HR was approximately 307 bpm. Most drugs administered, either in i.v. bolus (indomethacin, sulfisoxazole, TEA) or in i.v. perfusion (L-694,247, 1-PBG), did not modify MBP. However, and probably due to its mechanism of action, i.v. bolus administration of losartan (a blocker of AT_1_ receptors) [33] transiently decreased MBP and i.v. administration of L-NAME (a non-selective NOS inhibitor) induced a persistent elevation of MBP [29]. In this line, i.v. infusion of TCB-2, a 5-HT_2A_ receptor agonist, significantly increased MBP and HR as previously reported for other 5-HT_2_ receptor agonists [7,18]. In fact, 5-HT_2A_ receptor activation is associated with vascular smooth muscle contraction, platelet aggregation, and tachycardia [7,34], which may account for alteration in MBP and HR after i.v. perfusion of TCB-2. TEA and 1-PBG modified HR by reducing or increasing it, respectively, as previously reported by us [18].

### 3.2. Indirect Mechanisms Involved in 5-HT Vascular Sympatho-Inhibition in Female Rats

The electrical stimulation of sympathetic outflow induced frequency-dependent increases in MBP, without variations in HR, as previously shown by us [18]. In males, the direct 5-HT effect at the vascular and renal level and also the indirect vascular/renal effects of serotonergic system modulating the sympathetic or non-adrenergic-non-cholinergic systems are mediated by either vasoconstrictor or vasodilator agents, such as Ang-II, NO or EDH, in different experimental models, regardless of whether their involvement varies depending on the vascular territory studied as well as the pathophysiological conditions [29,30,31]. The serotonergic sympatho-inhibitory action is mediated, both in female and male pithed rats, by 5-HT_1_ receptors; while in males the 5-HT_1D_/5-HT_1A_ receptor subtypes seem to be involved in the serotonergic reduction in sympathetic discharge [6,7,19], in female rats 5-HT_1A_ receptor activation is devoid of this action and only the 5-HT_1D_ receptors are responsible of this inhibitory effect, since L-694,247 is able to reproduce 5-HT inhibitory effects on electrically induced vasoconstrictions (current data [18]).

Considering that 5-HT_1D_ receptor activation is coupled to G_i/o_ family proteins, which leads to (1) the decrease in cAMP levels, acting as a neurotransmitter inhibitor and (2) the stimulation of other signaling pathways, suggesting a role in the modulation of various cellular functions [35], we tried to determine the involvement of several vascular relaxing factors such as (i) NO, which is produced through the enzymatic conversion of L-arginine by NOS, (ii) prostacyclin via the COX pathway, and (iii) EDH, which usually promotes relaxation by hyperpolarizing vascular smooth muscle cells, involving the activation of K^+^ channels [36,37]. To do so, we intravenously pretreated the female pithed rats with either L-NAME [29,30], indomethacin [9,30,31], or TEA [38], prior to the i.v. perfusion of L-694,247 to evaluate if the presence of these mediators were implicated in the 5-HT_1D_ receptor-mediated vascular sympatho-inhibition. It is important to remark that in our study, in female pithed rats, TEA decreased per se the vasopressor responses induced by stimulation of sympathetic outflow, which is in contrast with data reported in male pithed rats where the non-selective K^+^ channel blocker TEA had no effect on the vasopressor responses elicited by sympathetic stimulation [38]. On the contrary, L-NAME, as already reported [29,39], a non-selective NOS inhibitor, enhanced the MBP increases evoked by electrical stimulation, which may result from the inhibition of relaxation normally caused by NO. Moreover, it has been demonstrated that the absence of NO leads to greater NA release from sympathetic nerves [40].

Intravenous administration of TEA completely blocks the L-694,247 vascular sympatholytic effect. The K^+^ channels are integral to the function of EDH [41] and the blockade of these channels by TEA inhibits EDH-mediated responses, underscoring their critical role in vascular tone regulation [42]. Thus, in female rats, the EDH pathway appears to mediate the inhibition of sympathetic neurotransmission via 5-HT_1D_ receptors (current data). These findings are partially in agreement with previous works in male rats treated with sarpogrelate, a 5-HT_2_ receptor antagonist, where the sympatholytic effect of 5-HT_1/7_ receptors was also mediated by endothelial factors. However, the sex, experimental model, and pathophysiological conditions seem to be critical in the involvement of specific endothelial pathways. For instance, a chronic 5-HT_2_ receptor blockade in male rats unmasked a COX-dependent mechanism (mainly COX-2) in the 5-HT_1D_ effect, whereas the 5-HT_7_-mediated sympathetic inhibition was produced through EDH [31]. Similarly, the induction of a diabetic state in male rats revealed the NO pathway as the main mediator of 5-HT_1A_ sympatho-inhibitory effects [29]. Moreover, our current results are consistent with previous studies suggesting that the EDH pathway plays a more prominent role in female rats [43,44]. Estrogen activity may underlie this sex difference in the prevalence of EDH responses. Indeed, an estrogen deficit in animals, achieved by ovariectomy, reduces EDH responses induced by several different stimuli and this effect is reversed by treatment with 17β-estradiol [43,45,46].

### 3.3. Indirect Mechanisms Involved in 5-HT Vascular Sympatho-Potentiation in Female Rats

We have also shown in female rats that the serotonergic system is also responsible for a sympathetic potentiation at the vascular level, through 5-HT_2A_ and 5-HT_3_ receptor activation (current data [18]). Taking into account that (1) direct serotonergic vasoconstrictor effects, through 5-HT_2_ receptor activation, at the renal level, are mediated through Ang-II in male rats [2] and (2) the induction of diabetes in males modifies the mediators involved in 5-HT_2A_-induced renal vasoconstriction, unmasking the COX pathway as the main actor [3]; we decided to evaluate in female rats the possible role of endothelium-derived contracting factors (Ang-II, prostanoids or endothelin) in the 5-HT_2A_ and 5-HT_3_ sympatho-enhancing action at the vascular level, administering i.v. losartan, indomethacin, or sulfisoxazole prior to intravenous infusion of TCB-2 (5-HT_2A_ agonist) or 1-PBG (5-HT_3_ agonist). None of these antagonists modified per se the vasoconstrictions obtained by electrical stimulation of sympathetic outflow.

Regarding the sympatho-excitatory effect induced by 1-PBG, this action was found to be independent of indirect pathways, as the blockade of COX, ET_A_, and Ang-II pathways did not modify the potentiation of vasopressor responses evoked by electrical stimulation during 1-PBG perfusion. It is important to remark that the 5-HT_3_ receptor is a ligand-gated ion channel, distinct from other serotonin receptors, which are typically G protein-coupled. Upon binding of serotonin to the 5-HT_3_ receptor, the channel opens to allow the influx of Na^+^ and Ca^2+^ ions, and the efflux of K^+^ ions, leading to membrane depolarization and rapid excitatory neurotransmission [47], which seems to form the basis of the direct vascular sympatho-excitatory effect in female rats (current data).

Concerning the sympatho-excitatory action induced by the activation of 5-HT_2A_ receptors, indomethacin and the ET_A_ receptor antagonist, sulfisoxazole, did not modify this effect. Unlike the blockade of AT_1_ receptors by losartan, it completely reversed the sympatho-potentiation induced by the 5-HT_2A_ receptor agonist TCB-2, exhibiting an increase in blood Ang-II concentration in females. The 5-HT_2A_ receptor is coupled to the G_q/11_ family, whose activation increases cytoplasmic calcium and induces phosphorylation of various downstream targets involved in cellular signaling [48]. It is probable that this cascade leads to a wide range of responses, including Ang-II release at the vascular level; in this context, our work shows that Ang-II levels are augmented following 5-HT_2A_ receptor activation in female rats. Although in male rats, 5-HT_2A_ is devoid of any vascular sympatho-excitatory effect [18], several studies have previously underscored the interplay between serotonergic and Ang-II systems at the cardiovascular and renal levels. In fact, at the cardiac level, it has been demonstrated that co-administration of 5-HT and Ang-II led to significant alterations in contractility and extracellular matrix remodeling of cardiac valve cells, enhanced cellular contractility, cytoskeletal reorganization, and increased collagen remodeling, that can contribute to valvular heart diseases [49,50]. Furthermore, at the renal level, we have demonstrated that direct 5-HT_2_ vasoconstrictor action is related to the AT_1_ receptor activation [2], confirming this synergistic effect. However, this work shows for the first time that the 5-HT_2A_ sympatho-excitatory effect at the vascular level is mediated by Ang-II in female rats.

### 3.4. Limitations and Clinical Perspectives

Our study has certain limitations that must be taken into account, as our pithed rat model eliminates all central nervous system control over blood pressure. Furthermore, our experimental design involving electrical stimulation does not allow for the direct measurement of norepinephrine release; instead, we rely on an indirect assessment based on the increases in blood pressure following stimulation. In addition, since NO and prostanoids also act via K^+^ channels, EDH-specific mechanisms could have been determined by dual NO/prostanoid inhibition (L-NAME + indomethacin) or selective EDH-pathway blockers (e.g., apamin/charybdotoxin). Nevertheless, as neither NO nor prostanoids separately alter the 5-HT_1D_ receptor-mediated inhibitory response, and only TEA (non-selective K^+^ channels inhibitor) reversed this serotonergic effect, we linked this sympatholytic effect to EDH.

Although CVD is the leading cause of death in the world, the cardiovascular risk factors seem to be quite different in women and men [21]. Thus, biological sex-based aspects appear to form the base of the clinical management pyramid for these disorders. In the 21st century, sex differences are extensively being studied in animal models, and mechanistic investigations should be undertaken to analyze these sex disparities, which in the end could lead to a sex-specific approach that may open a novel therapeutic avenue for CVD. The contribution of the sympathetic nervous system (SNS) to cardiovascular regulation has been widely studied and seems to be sex-dependent [51,52]; furthermore, currently there are commercialized pharmacological treatments that modulate SNS to reduce CVD, but with no sex or gender differentiation. Hence, there is still a great need to look for new sex-related therapeutic approaches to diminish morbimortality associated with CVD. Our findings provide a new insight into the intricate interplay between the serotonergic and sympathetic nervous systems at the cardiovascular level. The involvement of different mediators in this crosstalk (EDH or Ang-II) between males and females suggests a multifactorial mechanism that may contribute to sex-specific differences in cardiovascular homeostasis. Understanding these interactions could pave the way for the development of targeted and sex-tailored therapeutic strategies in the management of cardiovascular diseases.

## 4. Materials and Methods

### 4.1. Drugs Used

The drugs and the sources used in the research were sodium pentobarbital (Dolethal^®^; Vetoquinol; Madrid, Spain), sodium heparin (Rovi; Madrid, Spain), atropine sulphate (Scharlab; Barcelona, Spain), L-NAME hydrochloride, TEA chloride, d-tubocurarine hydrochloride, and 1-PBG (Merck Life Science S.L.U.; Madrid, Spain); L-694,247, TCB-2, sulfisoxazole, and losartan potassium (Tocris Bioscience; Bristol, UK); and indomethacin (Acofarma; Barcelona, Spain). The doses of all the drugs used were based on results from previous studies, and the infusion rate for the serotonergic agonists was 1 mL/h [4,5,18,31,38]. All the compounds were dissolved in saline at the time of experimentation, except indomethacin (dissolved in PEN).

### 4.2. Animals

Female and male Wistar rats (n = 75 and n = 5, respectively) aged 14–16 weeks with a body weight of 250 ± 25 g were obtained from the animal facility of the University of Salamanca. The animals were housed under controlled conditions in open-topped polycarbonate cages (4–5 animals per cage), at a constant temperature of 22 ± 2 °C, 50% humidity, 12 light/dark cycle, and received food and water ad libitum.

### 4.3. Surgical Procedure and Animal Preparation

The animals were anaesthetized with pentobarbital (60 mg/kg; i.p.) and a tracheal cannula was placed for artificial ventilation (1 mL air/100 g, 50 strokes/min; Harvard Rodent Ventilator, Model 683). A stainless pithing rod was inserted through the orbit and foramen magnum into the spinal cord. Jugular veins were cannulated for the continuous perfusion of agonists and for the bolus administration of antagonists/inhibitors (or their vehicles). The left carotid artery was cannulated and coupled to a pressure transducer, connected to an e-corder 410 amplifier (Model ED410, Cibertec, Spain) to record MBP and HR using Chart^TM^ (v5.5.11; eDAQ) and LabChart^TM^ (v7.2; ADInstruments) software. The stimulation of the entire sympathetic outflow was conducted using Cibertec Stimulator CS-9. One electrode was connected to the pithing rod, and the other electrode was inserted subcutaneously into a leg. To prevent thrombus, heparin (1000 UI/kg) was intravenously administered. The animals were treated with atropine (1 mg/kg) and d-tubocurarine (2 mg/kg) to avoid cholinergic effects and electrically induced muscular spasms, respectively [7,18,31].

### 4.4. Vascular Sympathetic Stimulation in Female Rats

When the animals were in a stable hemodynamic condition for at least 10 min, baseline values of MBP and HR were recorded. Afterwards, vascular sympathetic nerves were electrically stimulated as previously stated: 15 ± 3 V, monophasic pulses of 1 ms, for 25 s at increasing frequencies (0.1, 0.5, 1, and 5 Hz). An interval of approximately 5 min was maintained between each frequency. The responses obtained showed increases in MBP (∆MBP), without significant changes in HR, and basal MBP was restored immediately after interruption of the stimulation. The S-R curve E0 was achieved in 20 min [6,7,8,18,32].

**Figure 6:**
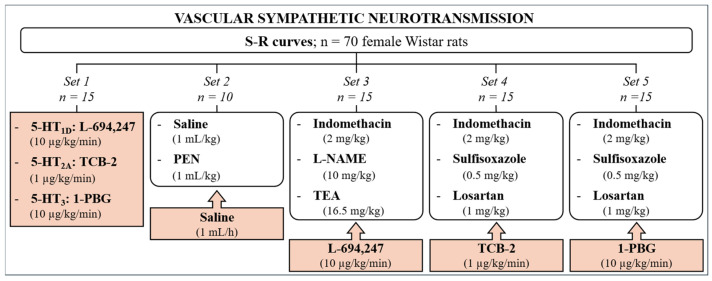
**Schematic representation of the in vivo experimental protocols and the number of animals used**. The scheme illustrates the five main experimental sets used in the vascular sympathetic neurotransmission study, where vasopressor responses were elicited by electrical sympathetic stimulation (S-R curves). 1-PBG: 1-phenylbiguanide; L-NAME: N(ω)-nitro-L-arginine methyl ester; PEN: mixture of 33% polyethylene glycol, 33% ethanol, and 34% NaOH 0.2 M; TEA: tetraethylammonium.

At this point, the animals were randomly divided into five sets (Figure 6). The first set (Figure 6) was performed to confirm the previous results [18]. The animals were administered a continuous i.v. perfusion of the selective 5-HT_1D_ (L-694,247; 10 µg/kg/min), 5-HT_2A_ (TCB-2; 1 µg/kg/min), or 5-HT_3_ (1-PBG; 10 µg/kg/min) receptor (sub)type agonist (n = 5 for each agonist). Then, two new E1 and E2 S-R curves were carried out equally to E0.

In the second set, the animals were administered i.v. saline (1 mL/kg) or PEN (1 mL/kg) (Figure 6; n = 5 for each vehicle). The corresponding curve (E0_saline_ and E0_PEN_) was completed after 10 min. Then, the animals received an i.v. infusion of saline (1 mL/h). After 10 min of starting the i.v. infusion, two new curves S-R (E1 and E2) were obtained as previously described for the S-R curve E0 (Figure 1).

In the third set (Figure 6; n = 15, using n = 5 for each antagonist tested) the animals were administered an i.v. bolus injection of, respectively, (a) a non-selective COX inhibitor, indomethacin (2 mg/kg), (b) a non-selective NOS inhibitor, L-NAME (10 mg/kg), or (c) a non-selective K^+^ channel blocker, TEA (16.5 mg/kg). The corresponding curve (E0_Indomethacin_, E0_L-NAME_ or E0_TEA_) was completed after 10 min, or 30 min in the case of L-NAME. Then, the animals received an i.v. perfusion of L-694,247 (5-HT_1D_ agonist; 10 µg/kg/min), and after 5 min, two new curves S-R (E1 and E2) were obtained.

In the fourth and fifth sets (Figure 6; n = 15 per set; n = 5 each antagonist tested) the rats received the following antagonists intravenously: (a) indomethacin (2 mg/kg), (b) an endothelin ET_A_ receptor antagonist, sulfisoxazole (0.5 mg/kg), or (c) an AT_1_ receptor antagonist, losartan (1 mg/kg), 10 min before its corresponding S-R curve (E0_Indomethacin_, E0_Sulfisoxazole_, or E0_Losartan_). After that, the animals received an i.v. perfusion of TCB-2 (fourth set; 5-HT_2A_ agonist; 1 µg/kg/min) or 1-PBG (fifth set; 5-HT_3_ agonist; 10 µg/kg/min), and two new curves S-R were obtained (E1 and E2).

### 4.5. Plasma Ang-II Determination

After the surgical procedure, in a group of n = 5, female rats’ blood samples were collected from the carotid artery before and 25 min after i.v. perfusion of the 5-HT_2A_ agonist, TCB-2. A group of n = 5 male rats was used to obtain blood samples after reaching a stable hemodynamic condition. Blood samples were collected into tubes containing heparin as an anticoagulant for plasma preparation and immediately centrifuged to separate the plasma (1000× *g* for 15 min at 4 °C) and stored at −80 °C until use. Plasma concentrations of Ang-II were measured using a rat Ang-II ELISA kit according to the manufacturer’s instructions, based on the ELISA sandwich technique. The ELISA kit for rat Ang-II was acquired from Cusabio (Catalog No. CSB-E04494r; Houston, TX, USA).

### 4.6. Data Presentation and Statistical Analysis

All experimental protocols and data analysis were randomized and blinded. Results are presented as mean ± SEM of at least five experiments (n = 5). The variations in MBP produced by electrical sympathetic stimulation are represented as increases in mm Hg from the baseline value. Statistical analyses were performed using GraphPad Prism 9.3.0 (GraphPad, USA). Normal distribution was determined using the Shapiro–Wilk test and homogeneity of variances was assessed by the Brown–Forsythe test. Changes in basal MBP and HR before and (i) during the infusion of serotonergic agonists or (ii) after i.v. administration of antagonists (or their vehicles) were evaluated by a *t*-test with Welch correction. Group differences using two variables (treatment and frequencies of stimulation) were assessed by two-way ANOVA, followed by Dunnett’s (compared to control group) post hoc test. For the biochemical assay, one-way ANOVA and Dunnett’s post hoc test (when comparing with female baseline levels of Ang-II) were employed.

Post hoc tests were conducted only if F in ANOVA achieved *p* < 0.05. Statistical significance was accepted at *p* < 0.05. Since the data obtained from the E2 curves onwards were practically identical, only the E2 curves are shown in the figures. In in vivo experiments (pithed rats), given that electrically induced increases in MBP in the presence of saline were similar to those produced in the E0 curve, the statistical analysis was performed versus saline.

## 5. Conclusions

This study demonstrates the involvement of indirect mechanisms in the serotonergic modulation of vascular sympathetic neurotransmission in female pithed rats. Our results suggest that the EDH pathway, via K^+^-channel activation, mediates the inhibition of sympathetic neurotransmission via 5-HT_1D_ receptors, while the sympatho-excitatory effect of 5-HT_2A_ receptors is associated with the Ang-II pathway. In contrast, the 5-HT_3_ sympatho-excitatory effect does not involve indirect pathways.

## Figures and Tables

**Table 1:** Increases in baseline values of mean blood pressure (∆MBP) and heart rate (∆HR) after 10 min i.v. bolus of antagonists (or 30 min after i.v. bolus of L-NAME) and i.v. infusion of 5-HT receptor agonists in female rats.

	Drug	Dose (mg/kg)	∆MBP (mm Hg)	∆HR (bpm)
**i.v. bolus** Antagonists	Indomethacin	2	0.7 ± 1.3	7.5 ± 9.9
	L-NAME	10	42.8 ± 12 *	0.3 ± 10.1
	TEA	16.5	−1.8 ± 1.2	−44.5 ± 6.7 *
	Sulfisoxazole	0.5	−3.6 ± 1.0	17.9 ± 13.3
	Losartan	1	2.0 ± 1.1	−5.7 ± 7.0
	**Drug**	**Dose (µg/kg/min)**	**∆MBP (mm Hg)**	**∆HR (bpm)**
**i.v. perfusion** 5-HT receptor agonists	L-694,247	10	−1.0 ± 1.9	16.0 ± 7.9
	TCB-2	1	30.2 ± 7.1 *	59.0 ± 11.7 *
	1-PBG	10	6.9 ± 1.3	90.0 ± 16.9 *

All values are expressed as mean ± SEM. Data were analyzed by *t*-test with Welch correction. * *p* < 0.05 vs. baseline; bpm: beats per minute. 1-PBG: 1-phenylbiguanide; L-NAME: N(ω)-nitro-L-arginine methyl ester; TEA: tetraethylammonium.

**Table 2:** Effect of i.v. bolus of antagonists/inhibitors on the increases in mean blood pressure (∆MBP) induced by sympathetic electrical stimulation in female rats.

i.v. Bolus Administration (mg/kg)	Frequency of Stimulation (Hz)			
	0.1	0.5	1	5
Nothing (control)	2.2 ± 0.3	10.5 ± 0.5	24.8 ± 2.3	54.1 ± 3.8
Indomethacin (2)	2.2 ± 0.2	10.3 ± 0.5	22.0 ± 1.5	52.4 ± 2.0
L-NAME (10)	4.8 ± 0.5 *	19.9 ± 1.2 *	37.6 ± 1.4 *	75.7 ± 3.1 *
TEA (16.5)	1.7 ± 0.1 *	7.2 ± 0.4 *	15.6 ± 0.7 *	39.7 ± 2.6 *
Sulfisoxazole (0.5)	2.2 ± 0.2	10.5 ± 0.8	21.9 ± 1.2	52.9 ± 2.8
Losartan (1)	2.4 ± 0.1	10.7 ± 0.4	22.5 ± 0.7	53.8 ± 2.1
	**∆MBP (mm Hg)**			

All values are expressed as mean ± SEM. Data were analyzed by two-way ANOVA followed by Dunnett’s post hoc test. * *p* < 0.05 vs. control. L-NAME: N(ω)-nitro-L-arginine methyl ester; TEA: tetraethylammonium.

## Data Availability

The main data are included in this manuscript. All data are available from the corresponding author on reasonable request.

## Abbreviations

The following abbreviations are used in this manuscript:

∆MBP	Increases in MBP
1-PBG	1- phenylbiguanide
5-HT	5-hydroxytryptamine, Serotonin
Ang-II	Angiotensin II
AT_1_ receptor	Ang-II type 1 receptor
CNS	Central nervous system
COX	Cyclooxygenase
CVD	Cardiovascular diseases
EDH	Endothelium-dependent hyperpolarization
HR	Heart rate
L-NAME	N(ω)-nitro-L-arginine methyl ester
MBP	Mean blood pressure
NO	Nitric oxide
NOS	Nitric oxide synthase
PEN	Polyethylene glycol (33%), ethanol (33%), NaOH 0.2 M (34%)
SNS	Sympathetic nervous system
S-R curve	Stimulation–response curve
TEA	Tetraethylammonium
